# Smelling Danger – Alarm Cue Responses in the Polychaete *Nereis* (*Hediste*) *diversicolor* (Müller, 1776) to Potential Fish Predation

**DOI:** 10.1371/journal.pone.0077431

**Published:** 2013-10-14

**Authors:** C. Elisa Schaum, Robert Batty, Kim S. Last

**Affiliations:** Scottish Association of Marine Science, Scottish Marine Institute, Dunstaffnage, Scotland; University of Genova, Italy, Italy

## Abstract

The harbour ragworm, *Nereis* (*Hediste*) *diversicolor* is a common intertidal marine polychaete that lives in burrows from which it has to partially emerge in order to forage. In doing so, it is exposed to a variety of predators. One way in which predation risk can be minimised is through chemical detection from within the relative safety of the burrows. Using CCTV and motion capture software, we show that *H. diversicolor* is able to detect chemical cues associated with the presence of juvenile flounder (*Platichthys flesus*). Number of emergences, emergence duration and distance from burrow entrance are all significantly reduced during exposure to flounder conditioned seawater and flounder mucous spiked seawater above a threshold with no evidence of behavioural habituation. Mucous from bottom-dwelling juvenile plaice (*Pleuronectes platessa*) and pelagic adult herring (*Clupea harengus*) elicit similar responses, suggesting that the behavioural reactions are species independent. The data implies that *H. diversicolor* must have well developed chemosensory mechanisms for predator detection and is consequently able to effectively minimize risk.

## Introduction

Predation is an important force that affects evolution, behaviour, life history and phenotypic traits in prey animals [1], [2] and animals in both the aquatic and the terrestrial environment have evolved predator evasion and avoidance mechanisms, which are not the result of direct attack. Many marine polychaete worms are involved in particularly interesting predator-prey interactions as they have to expose themselves to a relatively high level of risk every time they emerge from their sea-bed burrows [3]. At such times the energetic costs of not emerging to feed are outweighed by the costs of not being eaten by a predator [4]. Reliance on purely sound, mechanical disturbance and shadowing for predator detection would be of limited value due to the frequency of abiotic non-predatory stimuli in the marine environment [5]. Such abiotic cues are known to habituate nereidid polychaete worms and an argument has been made by [6] that the worms are likely to be in a state of almost permanent habituation to these stimuli.

Alternatively, prey can detect and responding to chemical cues which are diffusively carried in the water column and brought into polychaete burrows during irrigation bouts [7]. Substances triggering predator avoidance behaviour, hereafter referred to as alarm cues [8], [1], may be found in a variety of origins, such as predator mucous [9], skin cells [10], or the faeces, particularly in cases where the latter contains ingested prey items [11] [12]. Alarm cues may also be indirect, originating from other con-specific or heterospecific prey that have been attacked or injured [13]. Either has been described to have profound impact on prey behaviour in many invertebrate taxa, e.g. flatworms [14], brittle stars [15], zooplankton [16], [17] and gastropods [18], [19], but the exact pathways in which the recognition of these cues eventually leads to a behavioural response in marine polychaetes are not yet fully understood, despite the functional and ecological importance of this group [20,21].

It has been shown that another nereidid polychaete, *Platynereis dumerilii*, can modulate out-of burrow activity in response to chemical cues [22] and that the related king ragworm (*Nereis virens*) reduces its out-of-burrow activity and number of feeding events when exposed to con-specific whole-body homogenate [4]. Fish muscle extract also reduced the number of individuals feeding, but did not significantly lower activity levels, whereas extracts from the sympatric lugworm (*Arenicola marina*) and the congener (*Hediste diversicolor*) did not trigger any significant responses. Watson et al. [4] concluded that the whole-body extracts of conspecifics acted as an alarm signal, but it is unclear whether this chemical signal produced by an injured *N. virens* resembles that extracted from a whole-body homogenate or indeed would occur in detectable concentrations in the wild – in any case, distinguishing between conspecific and congeneric cues requires great levels of fine tuning [23]. The putative origins of the alarm cue compounds along with whether or not responses to alarm cues can be modulated in nereidids to adequately fit the cue’s concentration in the surrounding seawater have so far not been investigated. 

Fish mucous is one of the better described alarm cue candidates and has become known as a kairomone; an interspecific alarm cue affecting the recipient rather than the emitter [24]. Fish mucous is produced and sloughed off continuously and has a variety of functions in fish [25]. Studies suggest that it is probably not the mucous itself but its degradation products that act as kairomones [26]: In fish mucous, the active products are often disaccharide units released from the polymer by lyases of bacterial origin [27]. When fish were treated with antibiotics, significantly weaker predator-avoidance response in daphniids was found compared to mucous from untreated fish [27]. Further, Tvete and Haugan [28] showed that there is a metallo-protease component in mucus from plaice (*Pleuronectes platessa*) that kills mucus-dwelling bacteria, thereby reducing kairomone production. Other experiments [29] indicate that species-dependent effects on prey behaviour are due to different kairomone cocktails rather than differences in a single active ingredient.

### To assess whether *H. diversicolor*

[30] is behaviourally responsive to alarm cues associated with the presence of predatory species, we assayed out-of-burrow behaviour in *H. diversicolor* in response to first, juvenile flounder (*Platichthys flesus*) conditioned seawater; second, flounder mucous; third, mucous from another demersal juvenile flatfish, plaice (*Pleuronectes platessa*) and last, pelagic herring (*Clupea harengus*), which *H. diversicolor* would not usually encounter in the wild. *P. flesus* and *P. platessa* are both known to prey on ragworms [31] both at night and during the day with the incoming tide, whereas *C. harengus* does not. Juvenile demersal flatfish generally rely strongly on chemoreception for prey detection and feeding, but hunting for prey also depends on visual cues in plaice [32]. 

This study was carried out using video cameras with infrared illumination, linked to computers with motion detection software to record and quantify the following out-of-burrow aspects of polychaete behaviour: number of emergence events, distance, and emergence duration. Response variables were measured in order to determine the source, amplitude and threshold of a putative alarm cue. 

## Methods

### Ethical Notes

No specific permissions were required to sample on the shores of Loch Gilp, as the area is open to the public and not protected. Animal collection did not involve endangered or protected species. For detailed animal husbandry please see below.

All experiments were reviewed and approved by the SAMS ethical review committee. While commercial beam trawling is known to disrupt sediment flora and fauna, the hand-held beam trawl used here has an alloy frame and a mouth of no bigger than 1m and can be lifted with one hand. It causes less damage to the muddy sandy seabed than bigger, heavier trawls usually used in commercial fisheries or even the imprints left by digging by hand. We chose this method in order to better be able to assess the predators *H. diversicolor* would experience in their natural habitat – manual collection would have been highly exclusive and biased towards bolder predators. We collected fish this way twice. No fish died during beam trawling or transport to the lab (in 20 litre buckets of seawater, by car). Fish densities per stock tank never exceeded 10 fish, and fish were kept at this maximum density for no longer than a week. As all fish were juveniles weighing less 13 grams these are low densities. 

Blue mussels that were used to feed the fish had been collected outside the laboratory in the Dunstaffnage bay at low tide and had been killed by being chilled and then frozen at -20 °C. After the experiments, polychaetes were transferred to a 120 litre stock tank with a thick sediment layer. To avoid re-using and potentially conditioning worms, they were not reintroduced to their original stock tank. Fish that had not been used were released at the shores of Loch Gilp. All the experiments conducted complied with current laws regarding animal welfare in the UK. For this study, we had a permit to work on live invertebrates (in this case, worms and molluscs) and licences to sample mucous from dead fish. 

### Animal Collection and Husbandry

*H. diversicolor*, juvenile *P. flesus* and juvenile *P. platessa* were collected in August and September 2008 from the Loch Gilp shore, Scotland, UK (56° 01’ 46.20”N, 05° 25’ 54.03.49”W; WGS 84 datum, 2.5 m above chart datum) by hand digging and using a light-weight hand-held beam trawl respectively. All animals were transferred to the Scottish Association for Marine Science (SAMS), where husbandry conditions were maintained to closely match the conditions at the field site (temperature 14 ± 1 °C, photoperiodic regime 14 hours of light and 10 hours of darkness). All polychaetes where maintained in 50-70 litre stock tanks with sediment floors and were regularly fed pelleted trout feed. Since maturity can affect activity levels [33] polychaete maturity was determined by assessing gamete development as described in [33]. Only immature, intact worms of approximately the same weight were used in this study. All fish were kept in isolation from the polychaetes to ensure there were no physical or chemical interactions. The fish were maintained in 120 litre stock aquaria with sediment on the ground and were regularly fed crushed blue mussels before, but not during, experiments. 

### Monitoring of Polychaete Behaviour

Activity in *H. diversicolor* was monitored using CCTV cameras in combination with the Motion Grab software to log out-of-burrow activity. During the experiments, polychaetes were kept in four aquaria, each consisting of eight artificial burrows as described by Last [34,35], where between burrow spacing simulated low density populations for ragworms [36]. The aquaria were supplied from separate 210 litre reservoir aquaria and particular care was taken that all seawater supply to the various tanks was maintained in a flow-through state, which was constant between the treatment and control aquaria.

Polychaete activity was observed from above using infrared sensitive CCTV cameras (Sunkwang Electronics Co., Ltd.). External infrared light sources were used (Tracksys Ltd, UK) to allow constant recording both in visible light (day-time, ~ 0.004 candela) and constant, very dim light (night-time, ~ 0.00005 candela, to avoid noise from ambient light). Activity was logged with video capture cards (Leadtek Winfast TV2000 XP Expert TV) and the MotionGrab computer program [37]. This spatial actograph program can detect motion in multiple trigger zones, log that activity in text files and record frames in an AVI file. In this case, two trigger zones per burrow were selected, their position based on trials to ensure only one polychaete’s activity per burrow was included, and frames logged in one-second intervals. Video was subsequently analysed using the software MotionReadX [37] in order to quantify number, distance and duration of emergences (see also Figures S5 and S7). 

### Bioassay 1: Responses to Flounder Conditioned Seawater

Polychaetes were transferred to the experimental tanks and given two days to acclimate. Eight flounder (mean weight of 10.6 ± 0.2 g, surface area 14.76 ± 2.6 cm^2^) were transferred to a 210 litre tank which acted as the reservoir tank and supplied the two treatment aquaria, whilst the two control aquaria were supplied by a reservoir tank without fish. Air stones were used in both the treatment and control reservoirs to ensure water was oxygenated thoroughly and any potential alarm cue was well mixed. The flow-through rate for both the treatment and the control reservoirs was maintained at 72 ± 0.25 l/h per hour. All seawater used here was supplied from a beach with sub-sand intake filtration. Particulate organics in the water were minimal with no evidence of phytoplankton. Activity was monitored in all the worms (n=32) for a period of seven days. Following this predator conditioned seawater was diluted by reducing the number of flounder in the reservoir tank sequentially from six to two fish while all other parameters remained unchanged. Whenever fish numbers were reduced, the experiment was stopped. All fish were removed, in order to empty and scrub the experimental tanks with seawater, thereby removing as much predator cue as possible. In all essays, worms were only used once to avoid repeated measures and potential behavioural conditioning. 

### Bioassay 2: Responses to Mucous Conditioned Seawater

To determine if the alarm cue was derived from fish mucous, four *P. flesus* (mean weight 12.69 ± 0.24 g, mean surface area 19.32 ± 2.1 cm^2^) mucous was sampled immediately after death from fish killed by crushing the cranium. For sampling, we used the flat side of disposable surgical steel blades as this had proven to be the most efficient way of mucous sampling. Mucous was pooled and transferred to pre-weighed Eppendorf® test tubes and stored at -20 °C until required. For the assays, mucous was diluted using 0.2 µm-filtered seawater and 1 ml of the final solution was pipetted directly into the experimental tanks and mixed. Of the final eight dilutions, each was tested on at least two different tanks and on at least three different days (concentrations mentioned in the results section are as pipetted into the tank). During activity loggings, the seawater supply to the experimental tanks was turned off to ensure that mucous and its degradation products were not lost. Turning the water off on its own does not significantly alter any of the monitored behavioural traits in *H. diversicolor* (comparing overall activity levels in control worms: F _1,234_ = 1.15, p > 0.1). To determine if the alarm cue was present in mucous from other species, mucous was also sampled from juvenile plaice *P. platessa* and from adult herring *C. harengus*. Animal collection, husbandry and mucous sampling procedure for plaice were the same as for Bioassay 2. *C. harengus* was bought locally, having been caught and killed within 12 hours.

### Statistical Analysis

MotionRead data was extracted to provide number, duration and distance of worm emergences. For statistical analysis, these behaviours were summed for all animals in one tank due to their non-independence. Only night-time data was used for analysis since animals have been found to be largely nocturnal with very little out-of-burrow activity during the day [34], see also Material and Methods S1, Figure S1 and Figure S2. Normality of residual noise, (Kolmogorov-Smirnov test) and homogeneity of variances (Levene’s test) were tested prior to performing ANOVAs imbedded in a GLM in Minitab (version 15, Minitab Inc. UK) or lme in R (Version 2.10.1). For post hoc pair-wise comparison, the Tukey test or the Holm-Sidak test were used. More detailed methods including information on the statistical models used for analysis can be found in the Material and Methods S1, Reference S1. 

## Results

### Bioassay 1: Behavioural Responses to Flounder Conditioned Seawater

**Figure 1:**
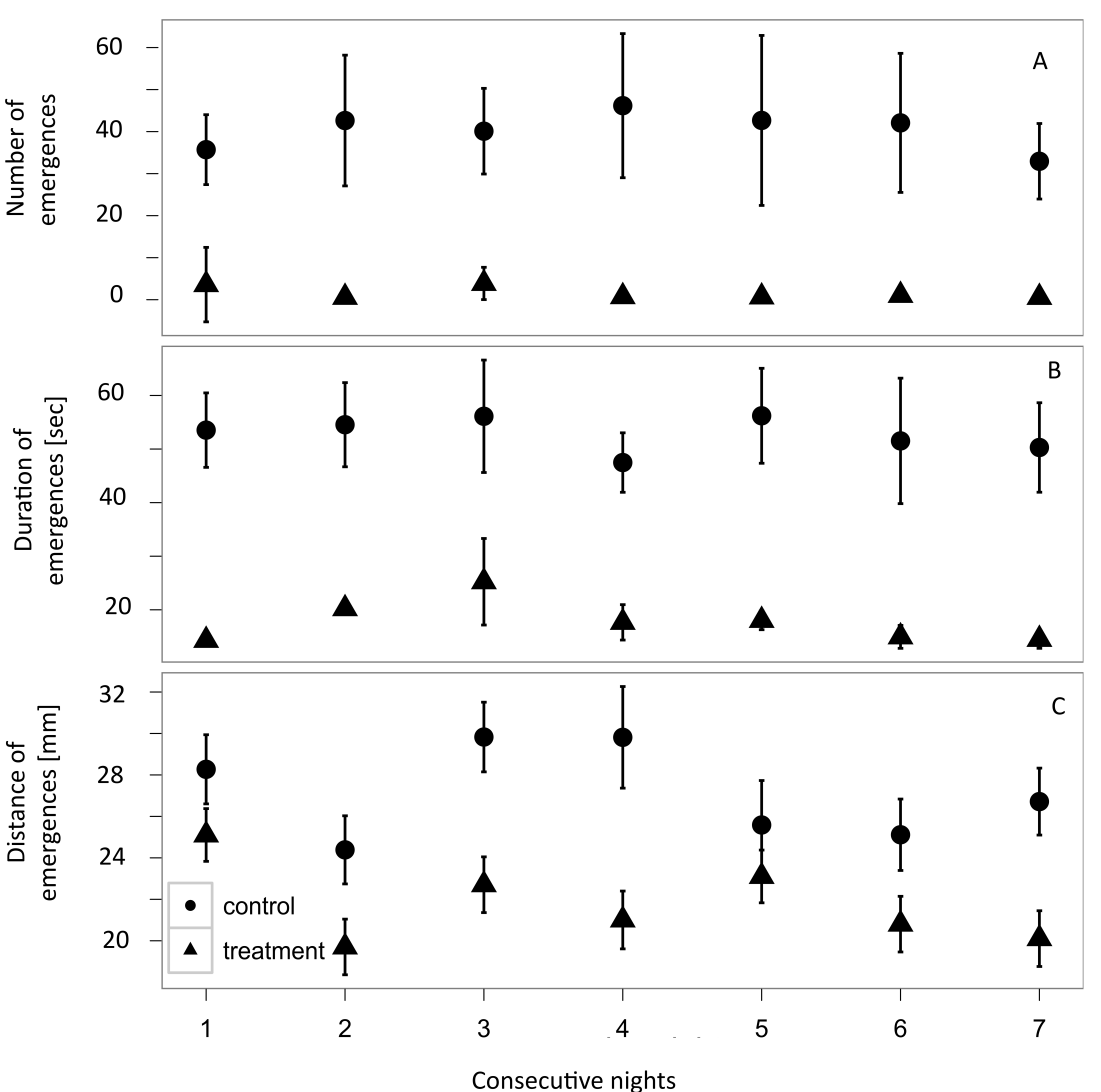
Out-of-burrow activity in the polychaete *Hediste* *diversicolor* in response to flounder (Platichthys flesus) conditioned seawater (triangles) is reduced compared to seawater control (circles) over a seven-night period : With eight juvenile predatory *P*. *flesus* in a reservoir tank supplying the polychaete tanks, all monitored traits of out-of-burrow behaviour in *H. diversicolor* were significantly reduced compared to a seawater control. a) Mean number of emergences per night is (average across all nights) reduced by 97 % (F _3,25_ = 37.8 p < 0.005); b) mean duration of emergences is, on average, reduced by 74 % (F _3,25_= 51.5 p< 0.005) and; c) mean maximum distance of emergences is reduced by 21% (F _3,25_= 25.5 p< 0.05). Recordings were made over 7 consecutive nights (dark phase 10 h). N=16 per treatment. Displayed are means ± one standard deviation.

**Figure 2:**
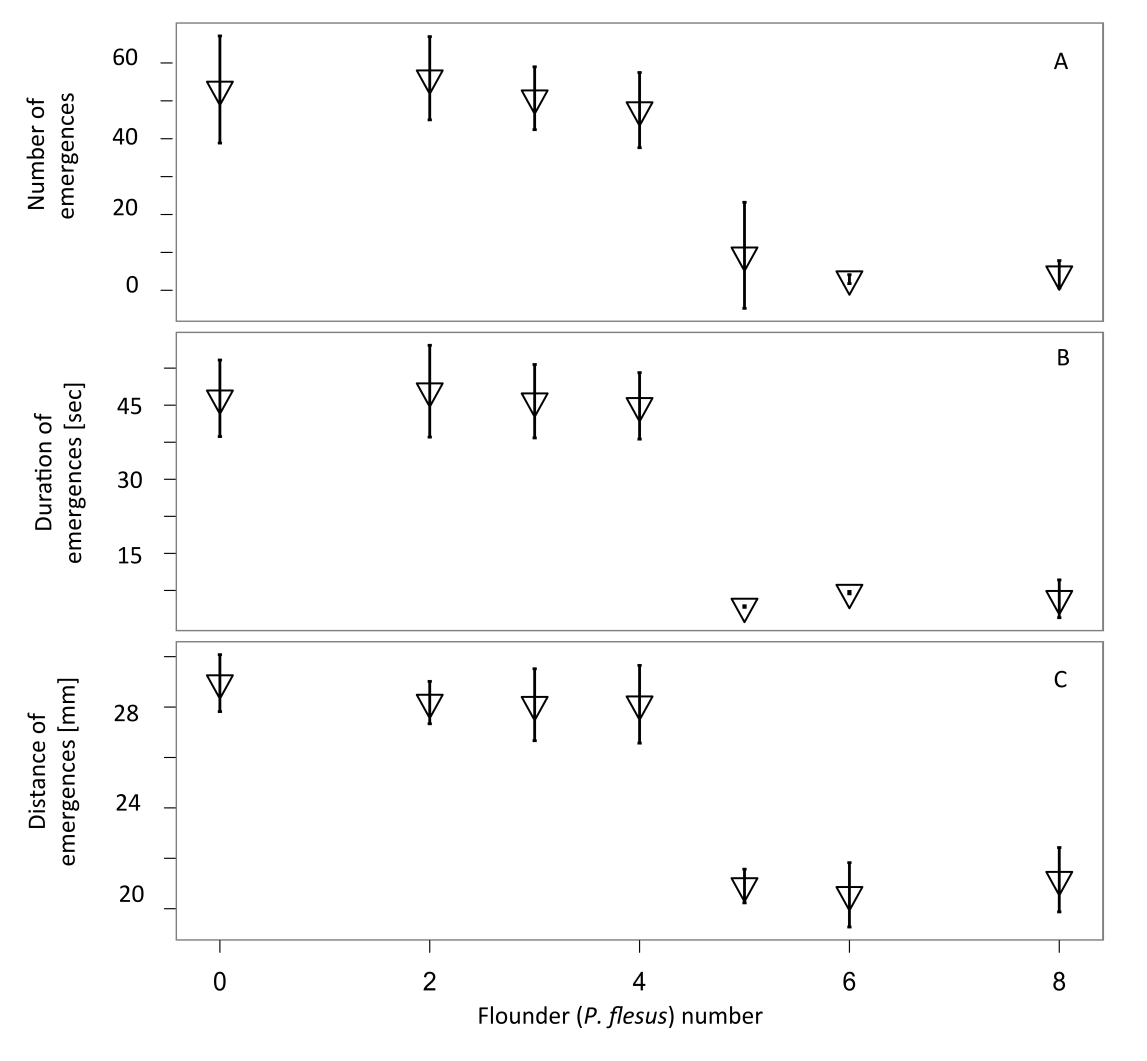
There is a marked threshold in changes in out-of-burrow activity in the polychaete *Hediste* *diversicolor* in response to increasingly flounder (Platichthys flesus) conditioned seawater. For up to four predatory juvenile *P*. *flesus* in a water tank supplying the polychaete tank, monitored out of burrow behaviour was not significantly different from that found in control animals. For five or more predators in the supplying water tank, polychaete behaviour changed markedly (H. *diversicolor* were less active). a) mean number of emergences per night. Adding five or more significantly reduced the number of emergence events (from 31.5 ± 7.71 to 2.15 ± 0.48 b) mean duration of emergences was reduced (from 5.45 ± 0.2 seconds to 1.41 ± 0.15 seconds) when water was conditioned with five or more *P*. *flesus*; c) mean maximum distance of emergences changed markedly (from 2.55 ± 0.13 cm to 2.04 ± 0.07 cm) when there were five or more *P*. *flesus* in the supplying water tank. Recordings were made over 5 consecutive nights (dark phase 10 h). N=16 per treatment with means ± standard deviation.

Number, duration and distance of emergences in all experiments were significantly higher during nights than during days (F_22,97_= 37.8, p<0.05, see also Figure S1 and S2, with 96% of all activity occurring during the night. Animals receiving flounder conditioned seawater significantly reduced the number of out-of-burrow emergence events (F_6,29_ = 37.84, p <0.005) when compared with animals receiving the seawater control (Figure 1A). When these animals did emerge it was for significantly shorter periods of time (F_6,29_= 51.00, p<0.005) and over shorter distances (F_6,29_= 25.5, p<0.05, Figures 1B and 1C respectively). There was no significant reduction in number (F_6,29_ =1.1, p > 0.1), duration (F_6,29_ =2.3, p>0.1) and distance (F_6,29_ =1.2, p>0.1) of emergence events with ≤ 4 flounder present in the reservoir tank when compared with control seawater without fish (Figure 2 A-C). However when ≥ 5fish are present in the reservoir tank, a significant reduction in all monitored behavioural traits was observed (overall model out-put for predator number affecting number of emergence events: F_1, 49_= 150.7, p <0.001, emergence distance: F_1,46_ = 83.44, p<0.001, emergence duration: F_1,46_ = 142.7, p <0.001.).

### Bioassay 2: Behavioural Responses to Mucous Conditioned Seawater

**Figure 3:**
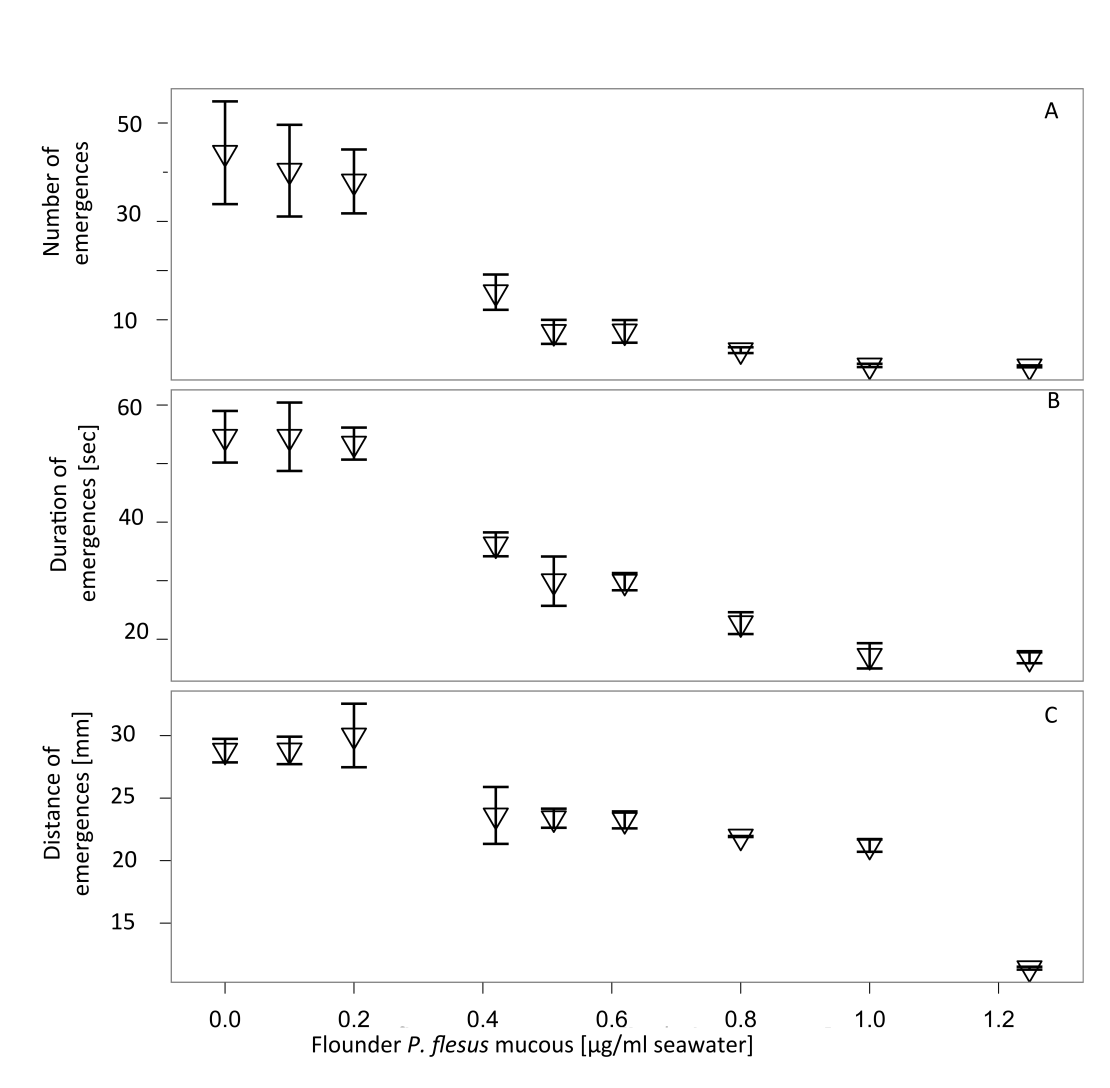
Out-of-burrow activity in the polychaete *Hediste* *diversicolor* decreases more gradually in response to increasing levels of mucous from flounder (Platichthys flesus) spiked sea water. We added mucous taken from *P*. *flesus* directly to the polychaete tank. Final mucous concentrations ranged from 0.1 to 1.2 µg per ml seawater. Behavioural changes in monitored traits in *H*. *diversicolor* occurred from a concentration of 0.4 µg/ml onwards and were generally more gradual than for the life predator experiments. a) Mean number of emergences for control and mucous concentrations below 0.4 µg/ml (40.33 ± 2.52) were reduced to 1.3 ± 0.18 for the highest amounts of mucous; b) mean duration of emergences was at its lowest for the two highest concentrations tested (1.38 ± 0.03 seconds); c) mean maximum distance of emergences: For mucous concentrations higher than or equal to 1 µg/ml, there was a significant reduction of foraging distance (average 1.55 ± 0.01 cm). Recordings were made over 3 consecutive nights (dark phase 10 h). N=16 per treatment with means ± standard deviation.

Compared to the sweater control, there was no significant reduction in number (F_7,34_=2.3 p > 0.1), duration (F_7,34_= 2.8 p > 0.1) and distance (F_7,34_= 3.1 p > 0.1) of emergence events when flounder mucous concentrations were smaller or equal to 0.2 µg/ml (see Figure 3 A-C respectively). For mucous concentrations of more or equal to 0.4 µg/ml a significant reduction in all monitored behavioural traits was observed (see Figure 3, overall model output for mucous concentrations ≥ 0.4 µg/ml: foraging Eevents: F_9,44_ = 57.6, p < 0.001, foraging duration F _9,44_ = 41.6, p < 0.001, foraging distance: F_9,44_ = 13.3, p < 0.05). 

**Figure 4:**
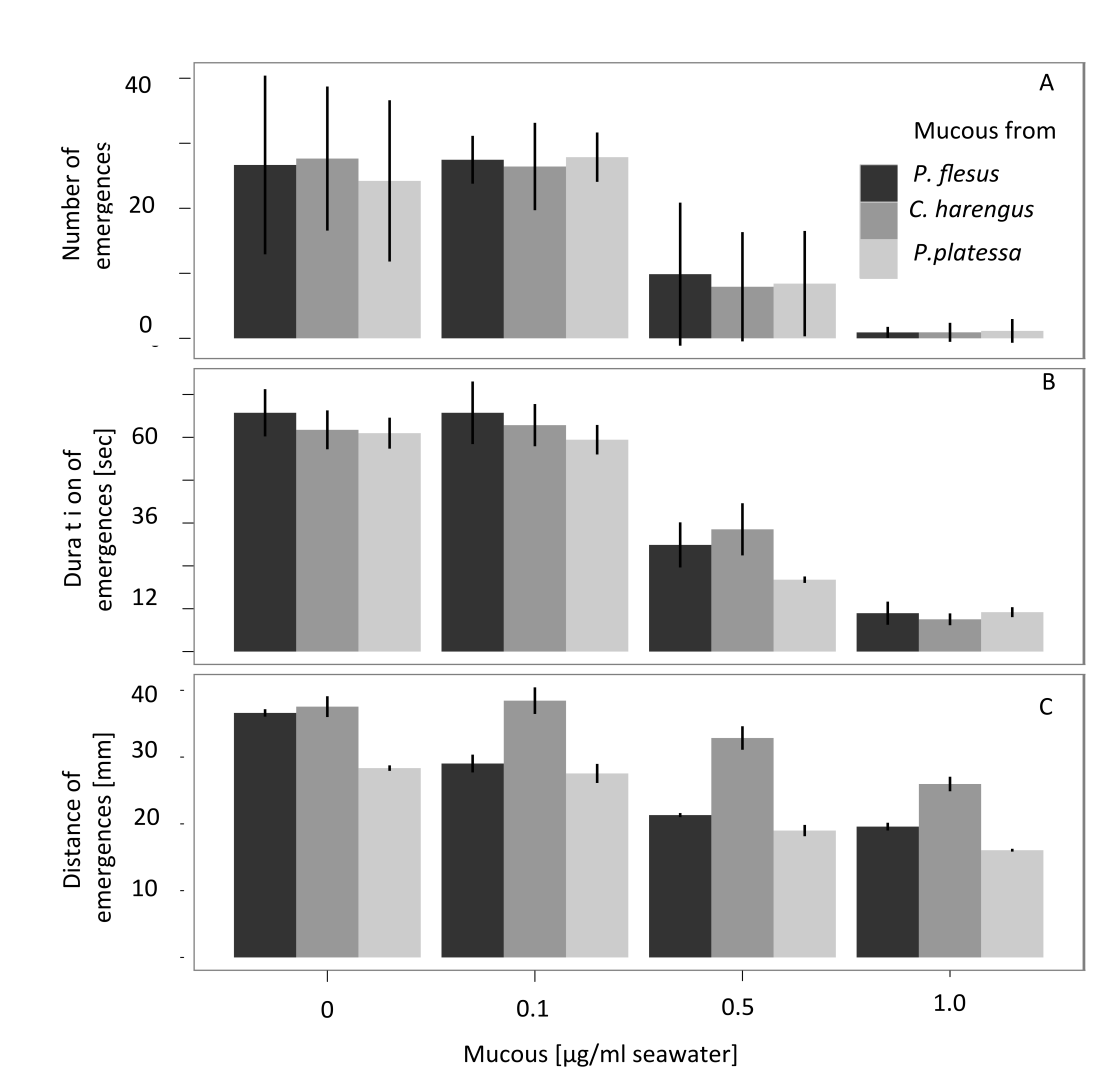
Most aspects of out-of-burrow activity in the polychaete *Hediste* *diversicolor* decrease in response to seawater spiked with increasing concentrations of mucous from flounder (Platichthys flesus), herring (Clupea harengus) and plaice (Pleuronestes platessa): a) mean number of emergences decreases with no significant difference between fish species; b) mean duration of emergences decreases. There are no significant differences between fish species; c) mean maximum distance of emergences decreases for all fish species used. However, the reduction in emergence distance in animals receiving water spiked with mucous from pelagic herring is significantly less marked. Recordings were made over 3 consecutive nights (dark phase 10 h). N=16 per treatment with means ± standard deviation.

Mucous from all fish species significantly reduced number, duration and distance of emergence events at concentration ≥ 0.5 µg/ml but not below (see Figure 4 A-C, over-all model output for any behavioural trait being affected by mucous from plaice and herring F _2,390_ = 235.79, p < 0.0001); there seemed to be no species-specific reaction (between species differences insignificant with F_2,388_ = 0.48, p > 0.5). This is backed up by a power analyses (see Figure S3 and Table S1). Mean emergence distance was less affected by herring mucous than by flounder or plaice mucous (Tukey post hoc test: p < 0.001). 

## Discussion

Here we have shown that the marine polychaete worm *H. diversicolor* is behaviourally responsive to a waterborne chemical cue(s), released by demersal as well as pelagic fish. All behavioural responses investigated, i.e. number, duration and distance of emergences from the burrow were reduced in the presence of predator conditioned or predator mucous spiked seawater above certain thresholds. Out-of-burrow food searching, which naturally exposes the polychaetes to predation risk [38], is therefore reduced and it is hypothesised that an unknown substance naturally occurring in fish mucous, or cocktail of substances [29], is acting as an alarm cue(s) to these worms. It is likely, that not all of the observed reduction in activity was a direct response to an alarm cue, but that a fraction also resulted from a positive feed-back loop: We assume that some worms will pick up the signal, reduce their activity, and release more cues themselves, which might in turn affect the behaviour of other worms in the same tank. As we have treated worms within one tank as not independent from each other in our analysis, it is likely that any such details are lost in the strong overall signal. More detailed research is needed as to how much – if any - information *H. diversicolor* can gather from alarm cues that do not originate from a directly attacked conspecific.

When *H. diversicolor* was exposed to a continuous seawater source containing the putative alarm cue from eight flounder there was no evidence of habituation over the seven-day trial. This is surprising for two reasons. First, mucous or alarm cue production by the fish is unlikely to have been constant over the entire time-course of the experiment, and second, habituation in this species has been documented, at least to stimuli such as changes in illumination, shadowing, mechanical shock and touch [5]. Evans suggests that habituation in nereidids must serve an important purpose since under natural conditions the worms are subject to almost continuous stimulation either from harmless or ‘lethal agents’. Habituation to chemical fish alarm cues has been documented in the freshwater isopod *Lirceus fontinalis*, where habituation in growth and leaf processing occurs after three days and it was suggested that the cue is only significant in eliciting short-term predator avoidance activity [39]. Since no habituation was observed in *H. diversicolor* we suggest that the alarm cue response must be strongly selective as animals failing to detect or respond appropriately are likely to be caught. Since our animals were never exposed to the actual predators during this set of experiments (note we ran trials with the predator within the actograph tanks on a different set of worms, see detailed methods in the Figure S8), the responses observed are either innate or learned as a consequence of associating the smell of predators with danger. Still, care must be taken when explaining these results, as mucous concentrations in either bioassay are likely to have been higher than they would in a natural environment. 

During a separate study (data not shown), we observed that worms which had undergone autotomy (loss of caudal segments) in the past were less active overall and showed a stronger response to predator alarm cues than intact animals. We tentatively suggest that they may have learnt by association as reported for other polychaetes and flatworms [14,40], but more details would be needed as to how the caudal segment was lost and to rule out that worms are less bold for others reasons (e.g. recovery). Since all animals in this study were intact with no evidence of recent caudal ablation, the observed response is more likely to be innate. 

Whilst the number and duration of emergence events reduced significantly in the presence of flounder conditioned seawater, the reduction in maximum distance from the burrow entrance was less obvious. *H. diversicolor* can retract into its burrow very rapidly due to a giant axon (or fibre) reflex response [41] and has a retraction speed of 17.3 mm s^-1^ with a response latency to sudden reductions in light and other stimuli ranging from 0.38 to 0.45 s^-1^ [5]. The time difference in retracting 10 mm (see Figures 1-4) will be very small and implies that, in order to minimise predation risk, distance may not be as critical as reducing event number and duration of out-of-burrow excursions.

Remaining in the burrow is an energetically costly method of predator avoidance. When in their burrows, *H. diversicolor* will be prevented from deposit feeding and burrow hypoxia will necessitate an increase in burrow seawater irrigation [7]. Observations from video data in our study show that animals that were not emerging as a consequence of the predator cue treatment were usually close to the burrow entrance (see Figure S4 for pilot studies with transparent tubes, where polychaetes could be seen irrigating the water close to the entrance, and Figure S6). This way, they avoid exposure to predators whilst minimizing burrow irrigation and still being able to sample the seawater column for food and as predatory cues [42]. Remaining in the burrow does not completely limit feeding ability in this species. If phytoplankton concentrations in the water column are high, particularly in stagnant water - [7] [43], *H. diversicolor* is able to filter feed by periodically ingesting a web of mucous, which it uses to catch particulate matter from the water column by active irrigation. This feeding strategy would greatly reduce the risk of predation, as has been suggested by [44]. Since the concentrations of mucous added to the tanks were low, and control animals did not become inactive, we assume that the observed changes in behaviour were indeed caused directly by an alarm cue, and not indirectly by a shift in organic matter concentrations or stagnancy of water. The cost-benefit trade-off between levels of phytoplankton or other organic matter required to illicit filter feeding and predator presence has not been experimentally demonstrated.

To determine if an olfactory/behavioural threshold in predator alarm cue detection exists in *H. diversicolor*, the numbers of predators in the conditioning tank were reduced. This revealed a threshold number of predators i.e. at least 2 grams flounder per litre, which significantly reduced the number, duration and distance of out-of-burrow events. This response threshold is interesting and suggests that the olfactory/behavioural pathways are either not graded, or that this assay is not sensitive enough to detect any graded response. Graded responses or predator-induced plasticity has been proposed as a concept by [45] and has, among other groups, been found in daphniids [46], copepods [9] and tadpoles [47], [48]. Other studies however have demonstrated an all-or-nothing response [1]. 

We hypothesised that the most likely source of alarm cue was originating from external fish mucous as has been found in other studies [9,49]. Indeed, serial dilutions of flounder mucous administered to the seawater of the experimental tanks, resulted in reduced emergence activities particularly between 0.2 and 0.4 µg/ml mucous in seawater. Unlike flounder-spiked water, flounder mucous does not show such a marked threshold alarm cue response, perhaps due to the more precisely graded mucous exposure employed in this assay. The implication is that *H. diversicolor* is able to detect predator abundance and modify their behaviour accordingly. 

The measured behavioural responses that minimise risk in these polychaetes are not solely restricted to mucous from flounder. There are also significant avoidance responses to demersal plaice (*Pleurenectes platessa*) and pelagic herring (*Clupea harengus*), suggesting that our findings are not species specific. This agrees with the findings of [50], who have shown that seawater incubated from four species of fish all elicited similar changes in diel vertical migration in brine shrimp, regardless of fish type. However our results, though all are significant, show that the weakest alarm cue response is to adult herring mucous. It is possibly, that the alarm cue cocktail in this species is different from that of demersal fish [51] or that pelagic fish emit lower alarm cue concentrations than demersal species altogether. In order to address this in detail, the active alarm cue ingredients and their concentrations in pelagic and demersal fish would need to be determined, but it is also likely that, albeit chilled, the alarm cues in *C. harengus* had already started to degrade [1], therefore eliciting a weaker response. 

Our finding that *H. diversicolor* must possess acute chemosensory ability is perhaps not surprising since its visual acuity is poor and restricted to shadow responses [5]. To our knowledge the processes involved in chemosynthetic predator detection in nereidids remains to be described (but see 41] [52] [53). Drawing from studies on other polychaete families, it would appear likely that the nuchal organs, parapodial cirri and feeding palps are most likely involved [54],

[55,56]. To better understand the underlying chemosensory mechanism and signal transduction pathways in predator detection first requires determining the active alarm cue(s) in nereidids which, in this case, have been shown to exist in fish mucous. 

Furthermore, it is likely that marine-predator prey interactions are bound to change along oceans that are being altered more rapidly than ever before 

[57,58]. Many recent studies have found that both predator [59] and prey [60] [61] will change significantly in how they learn to recognise, avoid and react to predators (or prey). We have shown that *H. diversicolor* would be a good model organism for such studies and that fairly high-throughput experiments can be conducted using the actograph /motion grab set-up, forming the basis for future studies concerning this ecologically important species. 

## Supporting Information

Figure S1 **Nocturnal behaviour of *H. diversicolor* under LD 14:10.**
(DOCX)

Figure S2 **A (control) and B (treatment – flounder conditioned sweater).** CLEAN spectral analysis – for periodicity.
(DOCX)

Figure S3 **Power analysis graphical output.**
(DOCX)

Figure S4 **Transparent actograph tubes, each housing one individual of *H. diversicolor*.**
(DOCX)

Figure S5 **Two *H. diversicolor* emerging from their respective burrows.** (Screenshot of MotionGrab data from 01/10/08).
(DOCX)

Figure S6 ***H. diversicolor* (red arrow) lingering at the entrance, but not emerging to forage (Screenshot of MotionGrab data from 01/10/08).**
(DOCX)

Figure S7 **Screenshots from MotionGrab file for the night of 10.10.2008 for control (seawater) and treatment (Pf conditioned seawater).**
(DOCX)

Figure S8 **Actograph Data (beam breaks per 30minutes) for a 7 –day trial where the predator *P. flesus* was in the actograph tank with the worms (white bars) and with manual agitation of the water (control, black bars).**
(DOCX)

References S1 **References for SI.**
(DOCX)

Material and Methods S1 **More detailed information on Motion Grab and Actograph.**
(DOCX)

Table S1 **Power-analysis output (using the pwr package in R) testing if the sample size used is sufficient.**
(DOCX)
