# Supplementary material for: Smelling Danger – Alarm Cue Responses in the Polychaete Nereis (Hediste) diversicolor (Müller, 1776) to Potential Fish Predation
Source: PLoS One. 2013 Oct 14;8(10):e77431. doi: 10.1371/journal.pone.0077431 (PMC3796461; doi:10.1371/journal.pone.0077431)
Supplement: Figure S1 — Nocturnal behaviour of H. diversicolor under LD 14:10. (DOCX) [file pone.0077431.s001.docx]

Figure S1

**Figure S1:** Nocturnal behaviour of *H. diversicolor* under LD 14:10. Replicates are tanks = Control =Treatment (water conditioned with 8 flounder).

, beam

* Significant ** highly significant differences between control and treatment as returned by post-hoc Tukey-test. Activity in controls (blue) is highest during the night for both control and treatment *H. diversicolor*. All means are displayed with positive standard deviations. A: Activity near burrow entrance as measured with Actograph set-up (Last 2003, 2009), beambreaks in 30 minutes B: overall activity outside of burrows (from raw Motion grab data: recording events above pixel threshold per 30 minutes).
