# Supplementary material for: Smelling Danger – Alarm Cue Responses in the Polychaete Nereis (Hediste) diversicolor (Müller, 1776) to Potential Fish Predation
Source: PLoS One. 2013 Oct 14;8(10):e77431. doi: 10.1371/journal.pone.0077431 (PMC3796461; doi:10.1371/journal.pone.0077431)
Supplement: Figure S2 — A (control) and B (treatment – flounder conditioned sweater). CLEAN spectral analysis – for periodicity. (DOCX) [file pone.0077431.s002.docx]

Figure S2

**Figure S2 A (control)** and **B** (**treatment** – flounder conditioned sweater) CLEAN spectral analysis. The main significant periods fall in the tidal range (~12.4 hours) and the lunar-day range (~24.8 hours). **C (control) and D (treatment):** Autocorrelation for data used in A. Both the tidal and the lunar-day peak found with CLEAN spectral analyses are above the 99% confidence interval. The strength of the lunar-day signal in particular is represented by the value of the 25-hour peak. Periodicity itself is not changed by the presence of predator signals. *H. diversicolor* remained nocturnal on a ~ 25 h cycle.
