# Supplementary material for: Smelling Danger – Alarm Cue Responses in the Polychaete Nereis (Hediste) diversicolor (Müller, 1776) to Potential Fish Predation
Source: PLoS One. 2013 Oct 14;8(10):e77431. doi: 10.1371/journal.pone.0077431 (PMC3796461; doi:10.1371/journal.pone.0077431)
Supplement: Figure S3 — Power analysis graphical output. (DOCX) [file pone.0077431.s003.docx]

Figure S3


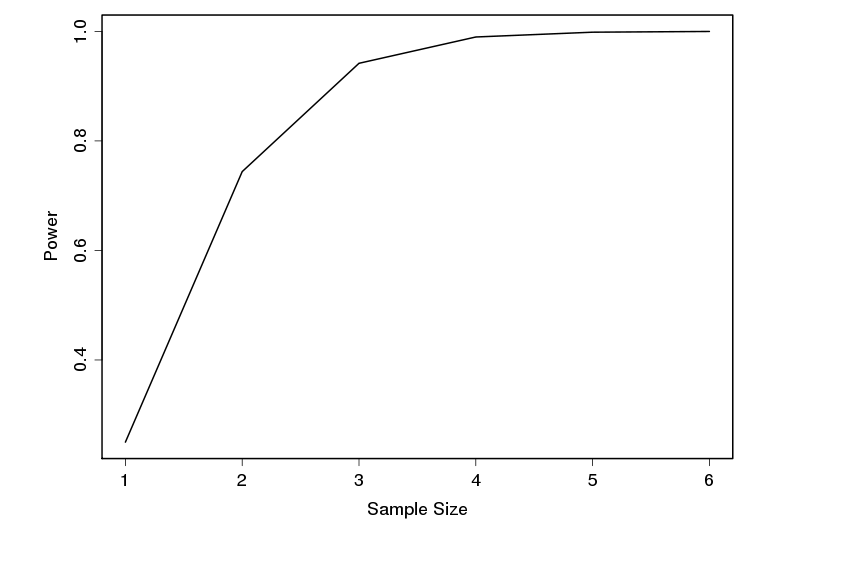


**Figure S3**: Power analysis graphical output. To reach acceptable statistical power, the number of predator species used must at least be three. Even higher statistical power can be achieved using 4 or more predator species (statistical power barely increases when more than 4 predator species are used).

“sample size” = number of predators used. “Power” = statistical power.
