# Supplementary material for: Smelling Danger – Alarm Cue Responses in the Polychaete Nereis (Hediste) diversicolor (Müller, 1776) to Potential Fish Predation
Source: PLoS One. 2013 Oct 14;8(10):e77431. doi: 10.1371/journal.pone.0077431 (PMC3796461; doi:10.1371/journal.pone.0077431)
Supplement: Figure S4 — Transparent actograph tubes, each housing one individual of H. diversicolor. (DOCX) [file pone.0077431.s004.docx]

Figure S4


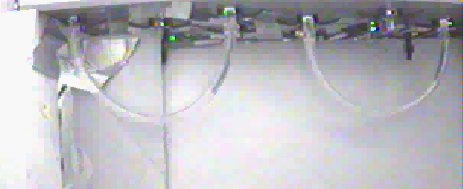

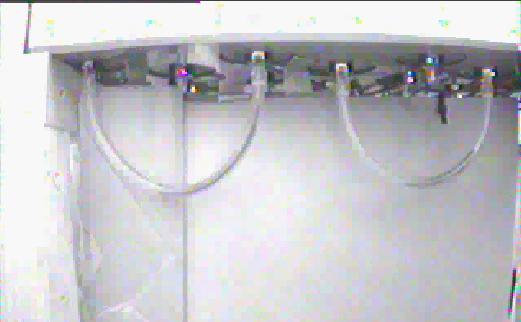

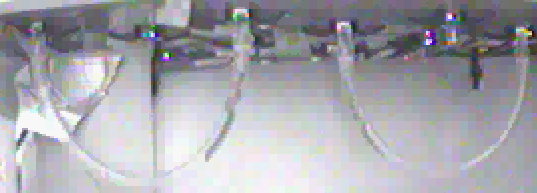


a

b

c

**Figure S4:** Actograph tubes, each housing one individual of *H. diversicolor.* Here, we used transparent tubes (in all other occasions black tubes were used) to better be able to assess behaviour near the entrance of the burrow (e.g. as a crude measure of increased irrigation near the burrow’s entrance when predators were present and food was scarce). Red lines indicate unbroken laser beam (no signal sent to computer). Absence of red lines indicates that the laser beam was broken and a signal sent to the computer.
