# Supplementary material for: Smelling Danger – Alarm Cue Responses in the Polychaete Nereis (Hediste) diversicolor (Müller, 1776) to Potential Fish Predation
Source: PLoS One. 2013 Oct 14;8(10):e77431. doi: 10.1371/journal.pone.0077431 (PMC3796461; doi:10.1371/journal.pone.0077431)
Supplement: Figure S5 — Two H. diversicolor emerging from their respective burrows. (Screenshot of MotionGrab data from 01/10/08). (DOCX) [file pone.0077431.s005.docx]

Figure S5:


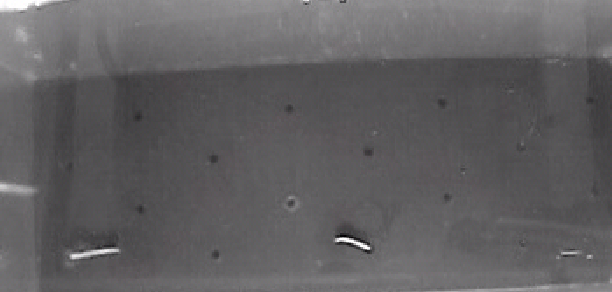


**Figure S5:** Two *H. diversicolor* emerging from their respective burrows. (Screenshot of MotionGrab data from 01/10/08)
