# Supplementary material for: Smelling Danger – Alarm Cue Responses in the Polychaete Nereis (Hediste) diversicolor (Müller, 1776) to Potential Fish Predation
Source: PLoS One. 2013 Oct 14;8(10):e77431. doi: 10.1371/journal.pone.0077431 (PMC3796461; doi:10.1371/journal.pone.0077431)
Supplement: Figure S7 — Screenshots from MotionGrab file for the night of 10.10.2008 for control (seawater) and treatment (Pf conditioned seawater). (DOCX) [file pone.0077431.s007.docx]

Figure S7

**Figure S7:** Screenshots from MotionGrab file for the night of 10.10.2008 for control (seawater) and treatment (Pf conditioned seawater). Each green line represents the maximal distance foraged during each foraging event. Numbers represent trigger zone numbers (not indicated in A for clarity).
