# Supplementary material for: Smelling Danger – Alarm Cue Responses in the Polychaete Nereis (Hediste) diversicolor (Müller, 1776) to Potential Fish Predation
Source: PLoS One. 2013 Oct 14;8(10):e77431. doi: 10.1371/journal.pone.0077431 (PMC3796461; doi:10.1371/journal.pone.0077431)
Supplement: Figure S8 — Actograph Data (beam breaks per 30minutes) for a 7 –day trial where the predator P. flesus was in the actograph tank with the worms (white bars) and with manual agitation of the water (control, black bars). (DOCX) [file pone.0077431.s008.docx]

Figure S8


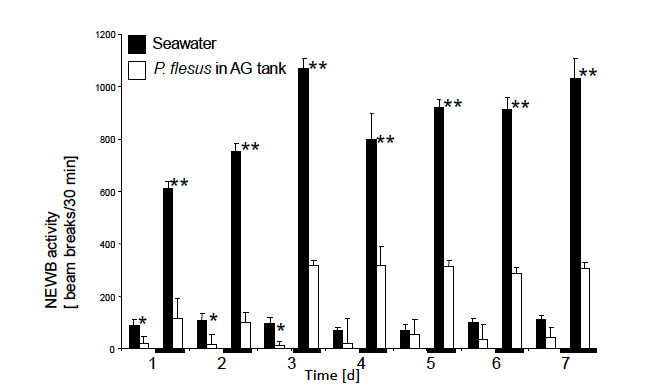


**Figure S8**: Actograph Data (beam breaks per 30minutes) for a 7 –day trial where the predator *P. flesus* was in the actograph tank with the worms (white bars) and with manual agitation of the water (control, black bars). Displayed are means and standard deviations. * significant difference between control and treatment, ** highly significant difference. Polychaetes were more active during the night than during the day, and presence of *P. flesus* significantly reduced activity measurable with the actograph set-up. Note that we could not use Motion Grab data for this trial as signal to noise ratio was poor due to the fish moving about the actograph tanks.
