# Supplementary material for: Smelling Danger – Alarm Cue Responses in the Polychaete Nereis (Hediste) diversicolor (Müller, 1776) to Potential Fish Predation
Source: PLoS One. 2013 Oct 14;8(10):e77431. doi: 10.1371/journal.pone.0077431 (PMC3796461; doi:10.1371/journal.pone.0077431)
Supplement: References S1 — References for SI. (DOCX) [file pone.0077431.s009.docx]

**SI References:**

**Cohen, J.** 1988 *Statistical Power Analysis for the Behavioral Sciences.* 2nd Edition. Lawrence Erlbaum Associates (USA)

**Last, K. S.** 2003. An actograph and its use in the study of foraging behaviour in the benthic polychaete, Nereis virens Sars. *Journal of Experimental Marine Biology and Ecology*, **287**, 237–248.

**Last, K. S., Bailhache, T., Kramer, C., Kyriacou, C. P., Rosato, E. & Olive, P. J. W.** 2009. Tidal, daily, and lunar-day activity cycles in the marine polychaete *Nereis virens*. *Chronobiology international*, **26**, 167–183.

**Rosato, E., Kyriacou, C.P**. 2006 Analysis of locomotor activity rhythms in *Drosophila*. *Nature Protocols* 1**(2),** 559-568
