# Supplementary material for: Smelling Danger – Alarm Cue Responses in the Polychaete Nereis (Hediste) diversicolor (Müller, 1776) to Potential Fish Predation
Source: PLoS One. 2013 Oct 14;8(10):e77431. doi: 10.1371/journal.pone.0077431 (PMC3796461; doi:10.1371/journal.pone.0077431)
Supplement: Material and Methods S1 — More detailed information on Motion Grab and Actograph. (DOCX) [file pone.0077431.s010.docx]

Table S1 Changes in soil pH in the different soil layers

|  | Soil pH | | | |
| --- | --- | --- | --- | --- |
|  | 0–10 cm | 10–30 cm | 30–50 cm | 50–100 cm |
| N addition |  |  |  |  |
| CK | 7.0±0.4^a^ | 7.4±0.3^a^ | 7.8±0.2^a^ | 8.1±0.1^a^ |
| N1 | 7.0±0.3^a^ | 7.5±0.3^a^ | 7.9±0.2^a^ | 8.2±0.1^a^ |
| N2 | 6.5±0.1^b^ | 7.3±0.3^a^ | 7.7±0.4^a^ | 8.1±0.2^a^ |
| N3 | 6.4±0.2^bc^ | 7.4±0.3^a^ | 7.6±0.5^a^ | 8.0±0.3^a^ |
| N4 | 6.1±0.3^cd^ | 7.3±0.4^a^ | 7.5±0.6^a^ | 7.7±0.9^a^ |
| N5 | 6.0±0.4^ce^ | 7.2±0.3^a^ | 7.6±0.4^a^ | 7.7±0.3^a^ |
| N6 | 6.0±0.4^de^ | 7.2±0.5^a^ | 7.6±0.3^a^ | 7.7±0.5^a^ |
| P addition |  |  |  |  |
| CK | 7.3±0.6^a^ | 7.4±0.2^a^ | 7.8±0.2^a^ | 8.2±0.2^a^ |
| P1 | 6.8±0.3^a^ | 7.5±0.5^a^ | 7.7±0.5^a^ | 8.1±0.2^a^ |
| P2 | 6.8±0.3^a^ | 7.4±0.2^a^ | 7.9±0.1^a^ | 7.8±0.3^a^ |
| P3 | 6.7±0.4^a^ | 7.6±0.2^a^ | 7.6±0.5^a^ | 8.1±0.2^a^ |
| P4 | 6.8±0.5^a^ | 7.3±0.4^a^ | 7.8±0.2^a^ | 7.9±0.2^a^ |
| P5 | 7.1±0.5^a^ | 7.4±0.3^a^ | 7.7±0.4^a^ | 7.9±0.3^a^ |
| P6 | 7.1±0.6^a^ | 7.5±0.4^a^ | 7.8±0.2^a^ | 8.0±0.5^a^ |

Data are represented as mean ± 1 SD (n = 6), and those designated with the same letters are not significantly different (*P <* 0.05).

Table S2 Changes in soil inorganic carbon (SIC) storage in the 0–100 cm soil layers

|  | SIC (g C m^–2^) | | | |
| --- | --- | --- | --- | --- |
|  | 0–10 cm | 10–30 cm | 30–50 cm | 50–100 cm |
| N addition |  |  |  |  |
| CK | 0.7±0.8^a^ | 40.4±38.8^a^ | 761.2±37.0^a^ | 3536.6±131.5^a^ |
| N1 | 0.7±0.6^a^ | 20.2±15.2^ab^ | 812.1±174.0^a^ | 3522.0±629.9^a^ |
| N2 | 0.4±0.6^a^ | 24.4±12.6^ab^ | 736.8±16.0^a^ | 3346.4±216.4^a^ |
| N3 | 0.4±0.3^a^ | 13.4±20.8^ab^ | 485.6±167.2^b^ | 3226.0±364.7^a^ |
| N4 | 0.7±1.1^a^ | 10.7±11.7^b^ | 372.2±135.4^b^ | 3492.9±233.0^a^ |
| N5 | 0.5±0.2^a^ | 14.9±17.9^b^ | 385.5±148.4^b^ | 3377.3±326.1^a^ |
| N6 | 0.6±1.0^a^ | 15.2±14.8^b^ | 512.9±249.7^b^ | 3186.1±583.4^a^ |
| P addition |  |  |  |  |
| CK | 3.6±4.2^a^ | 135.6±89.0^a^ | 1309.2±182.5^a^ | 2817.5±567.9^a^ |
| P1 | 9.7±9.5^a^ | 107.2±114.8^a^ | 1150.4±389.4^a^ | 3355.9±518.6^a^ |
| P2 | 5.2±6.1^a^ | 114.6±77.6^a^ | 1150.6±572.8^a^ | 2874.3±804.4^a^ |
| P3 | 3.9±6.1^a^ | 119.7±48.8^a^ | 1159.8±131.2^a^ | 3021.2±232.2^a^ |
| P4 | 6.2±8.4^a^ | 149.2±74.1^a^ | 1131.5±55.4^a^ | 2801.9±345.9^a^ |
| P5 | 5.4±8.6^a^ | 156.0±184.8^a^ | 1175.2±150.6^a^ | 2966.1±301.8^a^ |
| P6 | 6.5±7.2^a^ | 158.7±60.1^a^ | 1236.7±246.0^a^ | 2947.7±347.2^a^ |

Data are represented as mean ± 1 SD (n = 6), and those designated with the same letters are not significantly different (*P <*0.05).
