# Supplementary material for: Smelling Danger – Alarm Cue Responses in the Polychaete Nereis (Hediste) diversicolor (Müller, 1776) to Potential Fish Predation
Source: PLoS One. 2013 Oct 14;8(10):e77431. doi: 10.1371/journal.pone.0077431 (PMC3796461; doi:10.1371/journal.pone.0077431)
Supplement: Table S1 — Power-analysis output (using the pwr package in R) testing if the sample size used is sufficient. (DOCX) [file pone.0077431.s011.docx]

Table S1

Power-analysis output (using the pwr package in R) testing if the sample size used is sufficient to detect an effect of a given size with a given degree of confidence (i.e. determining the probability of detecting an effect of a given size with a given level of confidence, under sample size constraints as in our experiment). We tested if three predator species are enough to assume that in our experiment the effect of fish mucous on behavior in *H. diversicolor* is species independent. u = numerator degrees of freedom, v= denominator degrees of freedom , f= effect size (after Cohen 1988), sig. level=significance level. Power= statistical power. Power levels close to 1.0 are desirable.

| Multiple regression power calculation |
| --- |
|  |
| u = 9 |
| v = 132 |
| f2 = 0.15 |
| sig.level = 0.05 |
| power = 0.9029102 |
